# Making Bipedal Robot Experiments Reproducible and Comparable: The Eurobench Software Approach

**DOI:** 10.3389/frobt.2022.951663

**Published:** 2022-08-29

**Authors:** Anthony Remazeilles, Alfonso Dominguez, Pierre Barralon, Adriana Torres-Pardo, David Pinto, Felix Aller, Katja Mombaur, Roberto Conti, Lorenzo Saccares, Freygardur Thorsteinsson, Erik Prinsen, Alberto Cantón, Javier Castilla, Clara B. Sanz-Morère, Jesús Tornero, Diego Torricelli

**Affiliations:** ^1^ TECNALIA, Basque Research and Technology Alliance (BRTA), San Sebastián, Spain; ^2^ Spanish National Research Council (CSIC), Cajal Institute, Neural rehabilitation Group, Madrid, Spain; ^3^ Universidad Politécnica de Madrid, Madrid, Spain; ^4^ Optimization, Robotics and Biomechanics, Institute of Computer Engineering, Heidelberg University, Heidelberg, Germany; ^5^ Department of Systems Design Engineering, Department of Mechanical and Mechatronics Engineering, University of Waterloo, Waterloo, ON, Canada; ^6^ IUVO S. r.l., Pontedera, Italy; ^7^ Össur Iceland ehf, Reykjavik, Iceland; ^8^ Department of Biomechanical Engineering, Techmed Centre, Roessingh Research and Development, University of Twente, Enschede, Netherlands; ^9^ Center for Clinical Neuroscience, Hospital Los Madroños, Madrid, Spain

**Keywords:** software, benchmarking, replicability, exoskeleton, humanoid, algorithm, performance indicator

## Abstract

This study describes the software methodology designed for systematic benchmarking of bipedal systems through the computation of performance indicators from data collected during an experimentation stage. Under the umbrella of the European project Eurobench, we collected approximately 30 protocols with related testbeds and scoring algorithms, aiming at characterizing the performances of humanoids, exoskeletons, and/or prosthesis under different conditions. The main challenge addressed in this study concerns the standardization of the scoring process to permit a systematic benchmark of the experiments. The complexity of this process is mainly due to the lack of consistency in how to store and organize experimental data, how to define the input and output of benchmarking algorithms, and how to implement these algorithms. We propose a simple but efficient methodology for preparing scoring algorithms, to ensure reproducibility and replicability of results. This methodology mainly constrains the interface of the software and enables the engineer to develop his/her metric in his/her favorite language. Continuous integration and deployment tools are then used to verify the replicability of the software and to generate an executable instance independent of the language through dockerization. This article presents this methodology and points at all the metrics and documentation repositories designed with this policy in Eurobench. Applying this approach to other protocols and metrics would ease the reproduction, replication, and comparison of experiments.

## 1 Introduction

### 1.1 Context

Research on robotic systems is extremely active nowadays, and several platforms are now reaching the market. Nevertheless, the domain is still lacking in systematic tools for comparing the performances of these robots (Torricelli et al., 2015). From a research point of view, this lack of benchmarking tools results in studies that are complex to reproduce with new platforms or experimental protocols that cannot be easily replicated. The comparison of system performance is, therefore, either impossible or quite subjective.

The European project Eurobench addressed this issue by developing an integrated framework for enabling the systematic benchmarking of bipedal systems (humans, subjects using wearable devices, and humanoids) onto well-established experimental protocols and metrics. This framework consists of the creation of two benchmarking facilities, one for wearable systems and another for humanoids, where users could find testbeds and related protocols to benchmark their system (Torricelli and Pons, 2019). It also involves the creation of a centralized benchmarking software, accessible from a website. Interested persons can find there the list of protocols defined and connected to one of the facilities. The software is designed to allow users to upload data collected during experiments following one of these protocols and automatically obtain a set of performance indicators (PIs) computed on these data, which enable the comparison with other experiments and robotic platforms that have used the same protocol.

This article details the software methodology that has been defined for enabling such automatic and systematic benchmarking of experiments. The automation, which requires no manual intervention during the scoring process, is challenging in our context, as we did not want to constrain the programming language of the benchmarking algorithms used to generate the PIs. Instead of requiring the use of a unique programming language, we followed the strategy of constraining the interface of these programs, independent of the language, to come up with a common API for all, which is described in this document.

The article is organized as follows. The next subsection details related actions in the scientific community and positions the approach proposed by Eurobench. Section 2 describes the methodology used, and Section 3 highlights the developments that have been following this approach. Further, the conclusion section summarizes the characteristics of the approach selected and highlights some relevant further directions.

### 1.2 State of the Art

Benchmarking consists of comparing the performance of systems observed under similar experimental conditions following standardized testing procedures (Torricelli et al., 2020). Benchmarking is strongly intertwined with standards. Current developers of collaborative robots, such as exoskeletons or humanoids, have to face the challenge of certification (e.g., CE mark) if they want their product to be introduced in the market (He et al., 2017; Fosch Villaronga, 2018). Nevertheless, the availability of test methods for these technologies is scarce (Pinto-Fernandez et al., 2020; Massardi et al., 2022), and researchers typically use their own protocols to evaluate robotic abilities. Competitions like Cybathlon exist, wherein bipedal systems are challenged to perform similar tasks (Riener, 2016). However, the scoring metrics, such as the binary success or the execution time, do not permit characterizing and comparing precisely the performances of each system. Benchmarking can help in this process by enabling the plethora of methods available in the research arena to converge into a set of concrete standards. An example of this effort is the pre-standard CWA 17664:2021^1^ “Lower-limb wearable devices-performance test method for walking on uneven terrain,” developed by the Eurobench project.

If the benchmarking concept is simple, the reality is quite complex. In a recent study conducted with approximately 1,500 scientists, it is shown that 70% of these scientists have tried and failed to reproduce another scientist’s experiment, and 50% have failed to reproduce their own experiment (Baker’s study, 2016). In the same spirit, Kim et al. (2018) describe their journey in reproducing article results, even though the code and the dataset are available. In both these studies, the authors recommend internal peer review, better documentation, and method standardization, processes that may have a learning curve but would definitely make the difference later on.

Generating research that can be reproduced is a tendency that has been actively pursued in bioinformatics, a domain in which research involves significantly large datasets. Several journals propose to extend articles with sufficient material for reproducing the study. For instance, the journal Nature Protocols^2^ encourages authors to provide step-by-step instructions, and Plos^3^ requires authors to make available all data necessary to replicate the study. In addition, the platform protocols.io^4^, which is a framework enabling the creation, management, and sharing of experimental protocols and research methods, should be mentioned (Teytelman et al., 2016).

The importance of reproducibility has also been highlighted in the robotic community. Fabio Bonsignorio and his colleagues have conducted many activities to promote reproducible robotics, initially through the special interest group on good experimental methodology and benchmarking, Euron-GEMSIG^5^. Based on their work, the concept of reproducible research in robotics emerged (Bonsignorio, 2017), associated with guidelines for good experimental methodology (Bonsignorio et al., 2008). Since 2017, the IEEE Robotics & Automation Magazine has been supporting the publication of *R articles* reporting fully reproducible experiments. To achieve this, articles are to be provided with datasets, complete code, and hardware description. The framework codeocean^6^ is proposed to host datasets and related codes, to enable any interested reader reproducing the experiment seamlessly, a cloud tool enabling to store research material, share it, and even run code on online computing resources.

The arrival of cloud-based approaches, like codeocean or protocols. io, demonstrates that the technologies are getting ready. Likewise, several platforms are available for storing and sharing research data, such as Dryad^7^, figshare^8^, zenodo^9^, or OSF^10^. Nevertheless, they may not be providing material or low-level APIs for enabling benchmarking activity to occur in a systematic way, which is a challenging objective of the Eurobench initiative. The benchmarking aspect goes beyond the reproducibility process in the sense that through benchmarking scientists are not only interested in reproducing the results of a given experiment but also in comparing the performance levels measured across several experiments, involving or not different robotic platforms.

Whitaker (2017) and Kim et al. (2018) define the concepts of reproducibility, replicability, robustness, and generalizability. From a software point of view, reproducibility means obtaining the same results using both the same code and same data. Replicability is achieved if we can use the same code with different input data. Robustness and generalizability characterize the quality of the concept: the first one is obtained if the same result is obtained with the same data but different code, while the second is obtained if a different code and dataset are used. As will be seen in the next section, reproducibility and replicability are being looked at in the Eurobench framework. The reproducibility is considered mandatory to assess the stability of the metric implementation as well as ensure the code maintenance along time. The replicability is the concept required for enabling benchmark, as data coming from different experiments will be fed into the same algorithm to get comparable results.

## 2 Methods

To ease benchmarking experiments and result comparisons, Eurobench proposes to harmonize the experimental methodology. As stated by Remazeilles et al. (2022), all experimentation should be associated with a benchmarking protocol and a set of performance metrics. The benchmarking protocol documentation should describe the scope, purpose, and related requirements. This can be done through the definition of

(i) the related scenario, i.e., the functional task(s) to realize in a given environment;
(ii) the objective of the protocol, i.e., the description of the features the protocol aims to characterize;
(iii) the target subjects, if there exists exclusion and inclusion criteria;
(iv) the testbed equipment required to perform the experimentation;
(v) the PIs that will be used to score the behavior of the subject(s) and/or the wearable device;
(vi) the protocol procedure, describing all operations should follow the experimenter to conduct the experimentation. It can also define the appropriate number of repetitions, the setting(s) that should be changed to analyze the system behavior under different conditions, etc.

In Eurobench, we proposed a protocol template excel file^11^ to set all this information, but additional documentation should be provided to exhaustively describe the sensor placement, testbed structure, information that must be collected during the experimentation, etc. It is also important to state the performance metrics in the documentation, as it can be another criteria for the selection of a given protocol by the experimenter.

Altogether, the protocol description should permit the experimenter to drive the experimentation and collect the expected dataset.

**FIGURE 1:**
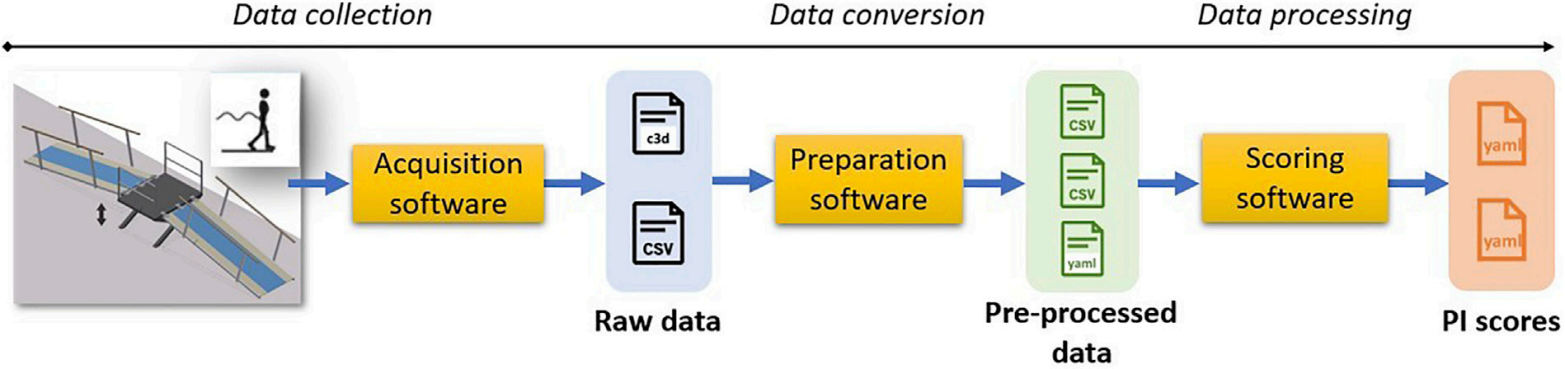
Segmentation of the software layers involved during an experimentation process, from data collection to experiment scoring.

Figure 1 highlights the successive operations involved from the experimentation start to the desired metric computation. Three different types of software are involved:

• the software needed to conduct the experiment and collect information (acquisition software);
• the software needed to prepare the collected data (preparation software);
• the software used to benchmark the experiment, i.e., to compute specific metrics based on the data collected (scoring software).

Following the terminology used in Figure 1, the *acquisition software* is dependent on the equipment used. In ideal, it should be provided with the protocol documentation or at least be sufficiently described to permit other experimenters to conduct similar acquisition with similar equipment. If an experimenter uses different equipment, he/she should document the testbed used to fully describe the captured data.

The *preparation software* aims to convert the collected data into a more generic structure, when appropriate. If the experimenter uses exactly the same setup as the one suggested in the protocol documentation, similar software could be used. If not, it is up to the experimenter to convert the collected data into the expected generic format.

The last block, the *scoring software*, contains all the algorithms used to calculate the PIs necessary to quantify the different abilities of a system. Thanks to the previous layer, the scoring software should be agnostic to the specific equipment used in the testbed. Thus, it should be able to process any experiment following the same protocol, described by similar datafiles, thereby ensuring the replicability, e.g., allowing the calculation using data collected on a different testbed.

The challenge is thus to enable another experimenter to conduct a similar protocol to collect the data, then prepare them, and finally use the very same algorithm to compute its associated scores, so that it can be compared with other experiments under the same protocol.

An additional challenge Eurobench is willing to tackle is to get this computation done automatically, independently of the protocol, the collected data, and the algorithm in charge of the calculation.

To attain this, it is necessary to define:

• a common terminology, to make sure all experimenters and protocol providers share similar concepts;
• the type of data that are collected from an experiment and how the collection of files is organized;
• a strategy to handle systematic approach algorithms possibly implemented in different languages.

### 2.1 Benchmarking Terminology

In a recent study, we defined terminology for concepts involved in experimentation (Remazeilles et al., 2022). Focusing on the items related to an experiment, the following terms can be highlighted.

A *run* is the recording of a single instance of the action requested of the subject (like walk on a slope and stop at the top). An experiment may contain several runs of the very same action for statistical purposes.

**FIGURE 2:**
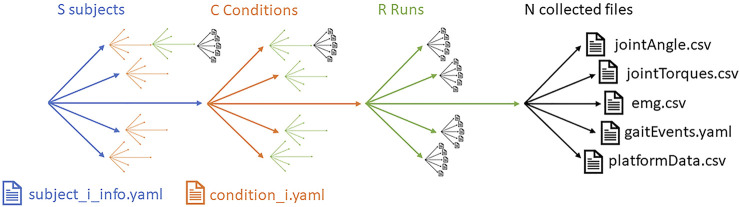
Data collected during experimentation.

*Controlled variables* are conditions and parameters that are needed to fully describe the experimental settings. They may refer to the configuration of the testbed (slope angle and length), tunings of the robotic device, indications given to the human subject, etc. These variables shall remain constant during a run execution and all the following repetitions. *Condition* is the term for the overall settings of these controlled variables. If several conditions are considered, the variations should be logged (we propose using a file named *condition_C.yaml* where C is the number of different settings), and for each condition setting, N runs should be performed (as illustrated in Figure 2).

During the experimentation, a set of datafiles gets collected. We distinguish the notion of *raw data* and *preprocessed data* (see Figure 1). By raw data, we refer to a datafile that may be defined in a proprietary format or using an ad hoc structure depending on the maturity of the data acquisition software.

To permit data exchange and comparison, it is a good practice to bring the raw data into a format agnostic to the brand of the sensor used for collection. Such sensor-agnostic data are named *preprocessed data*. Its format should be consistent independent from the software and hardware used to capture the information. The use of a preprocessed data format enables counterbalancing the potential differences in the testbed equipment and increases the replicability of the PI algorithm.

Distinguishing between raw data and preprocessed data also enables the handling, up to a point, of testbeds equipped with slightly different equipment. The preparation software should enable the experimenter to bring the collected raw data in the preprocessed format expected by the metric algorithm.

In Eurobench, we proposed a set of preprocessed datafile formats (see input data format in the online documentation^12^), where:

• datafiles are focused on a coherent set of measures or split into several files otherwise;
• the *.csv* format is encouraged for any periodic measure: each line is associated with a given timestamp, and the first line should name each column of information;
• for more punctual measures (like gait event or gait parameters), the YAML format is promoted.

The proposed datafile formats are described with their expected units. If a given protocol (or more exactly, a related metric) requires specific measures (like specific joint angle, measure units, acquisition frequency), such information should be stated in the protocol description.

We can relate our preprocessed data format choices with similar efforts found in the literature, in particular, in the research related to human motion analysis. In the study by Carfì et al. (2019), the University of Genoa created a multisensor dataset of human–human handover, composed of over 1000 recordings, where sensor information is stored in excel files as timestamped time series of 3D joint and marker coordinates. Maurice et al. (2019) presented the AnDyDataSet database related to industry-like manual activities. The collected data, available in proprietary or sensor-specific formats, are also provided in the csv format. The KIT motion database is another large-scale whole-body motion database of captured raw motion data as well as the corresponding postprocessed motion files (Mandery et al., 2015). Human motion is characterized using a set of 56 markers on the human body captured at a fixed frequency. The marker positions are stored using the standard c3d format and an original XML format, the *Master Motor Map*, which is proposed to ease the navigation in the timestamped files. In the study by Wojtusch and von Stryk (2015), the database contained not only the position of 35 markers recorded at a fixed frequency but also the electrical activity of 14 muscles from the legs and force plate sensing. The database and recording processes are well documented as well as the source code. The authors decided to store all datafiles as .mat files, matlab proprietary format, which can contain structured and hierarchical data information.

The distinction between raw and preprocessed data is thus observed in the literature. The usage of the csv format is also a common practice. Even though it may not permit to contain meta information on the data included, it presents the great advantage of being easily handled by any software language, and it is thus more likely to be accepted by the community and the algorithm providers. Regarding the internal file structure and field names, the proposed formats are based on discussions with different protocol providers, with whom we tried to reach a consensus. In any case, any format adjustment is acceptable as long as this adjustment is well documented in the protocol description.

### 2.2 Experimental Files

Experimentation may involve several subjects, requested to perform a given action and repeat it under different conditions. For consolidating the analysis, several repetitions or runs may be required for each conditional setting. The amount of collected information is significant, as illustrated in Figure 2.

All the data collected need to be organized. We propose the following structure:

• any file generated should be focused on a single run,
• a datafile should focus on a single type of information,
• the file naming should enable to distinguish the subject (X), condition (C), run (R), and file content.

We thus assume an experimental dataset to be composed of files like:

• subject_X_cond_C_run_R_dataType.{yaml, csv}
• subject_X_info.yaml
• condition_C.yaml

File condition_C.yaml should set the configuration values of any setting of the environment in a part of the experimentation, and subject_X_info.yaml should contain any relevant information about the human subject involved in the experiment (while respecting GDPR constraints). This information may be used by the scoring algorithms or just serve the purpose of documenting the experimental setting. The information contained in these two files may not be directly measured by the experimental setup.

The proposed naming for the datafile presents the advantage of being explicit. From its format, we can relate the subject involved (characterized in subject_X_info.yaml), the configuration of the settings (condition_C.yaml), the run number, and the data type it contains. The proposed file naming format also permits the direct deduction of the number of subjects (X), runs (R) or different conditional settings (C) applied in the experiment.

Some experimenters may argue that a folder-based structure would be better. Having such an explicit filename reduces the importance of folder organization, and in any case, switching from one format to another should just be a simple automation process.

It is a good practice to provide, together with the data collected (raw and preprocessed), some documentation files that will permit any reader (including the experimenter) to understand all collected data, in particular, if some deviations with respect to the protocol specification occur (in particular, if a different testbed acquisition device is used or if specific adjustments have been added for specific ad hoc studies).

### 2.3 Standardizing Algorithm Interface

The Eurobench project forced us to consider the harmonization or standardization of the PI algorithm, mainly for two reasons: 1) we wanted the computation to be automatic and 2) the protocol and algorithm providers were numerous.

To tackle the first item, we propose to standardize the metric algorithm interface (input and output) according to the following criteria:

(1) A PI algorithm should be launched using the data of a single run.
(2) All input files should be explicitly provided to the program as input parameters.
(3) The output of the program consists of a set of PIs, one PI per file.
(4) The generated output files are stored in a folder provided as the input parameter of the program itself.
(5) The program should be able to run in batch mode, i.e., without any interaction.

Item 1 presents the advantage of drastically simplifying the algorithm. As a single run is processed, there are fewer files to provide and less looping in the code (per user, condition, run, etc.). A simpler algorithm is easier to maintain and revise, and in the end, it is more focused on its real added value, which is computing the metric based on a single execution of the action.

With item 2, we avoid making any assumptions about the datafile storage organization and bring the program close to a classical standalone function, for which all required parameters are provided as input.

Items 3 and 4 are related to the results of the program or the output of the scoring function. Explicitly specifying the folder where the results should be stored permits to be independent of the way the program is launched. The algorithm does not make any assumption about where to store the results and just feeds the defined folder.

With item 5, we request algorithms to be implemented so that no human intervention is expected once the algorithm is launched. The algorithm should obtain all the information required to compute the metrics from the input files. This is of major importance if the algorithms are to be launched automatically.

To ease the automatic processing of the results, we also propose to harmonize the format of the output. As the PI score should be numerical information, we define the PI score as follows:

• each metric is stored in a YAML file, which is an open format;
• to be self-explanatory, a key “type” indicates the structure of the score;
• a key “value” is then used to indicate the score;
• as an alternative, a key “label” can be used to assign a name to the stored values, which is useful when the score is composed of various values.

Although, through the analysis of the different PIs proposed by the protocol providers, we observed that it is not always possible to reach the “Graal” metric, as simple as a scalar, where a high/low value would be defined as better or worse depending on the metric. We identified the need for storing more complex metrics, such as vector, matrix, list of vectors, and list of matrices. This means that the comparison of experiments based on complex metrics may require additional work (comparing matrices is not as trivial as comparing scalars).

**FIGURE 3:**
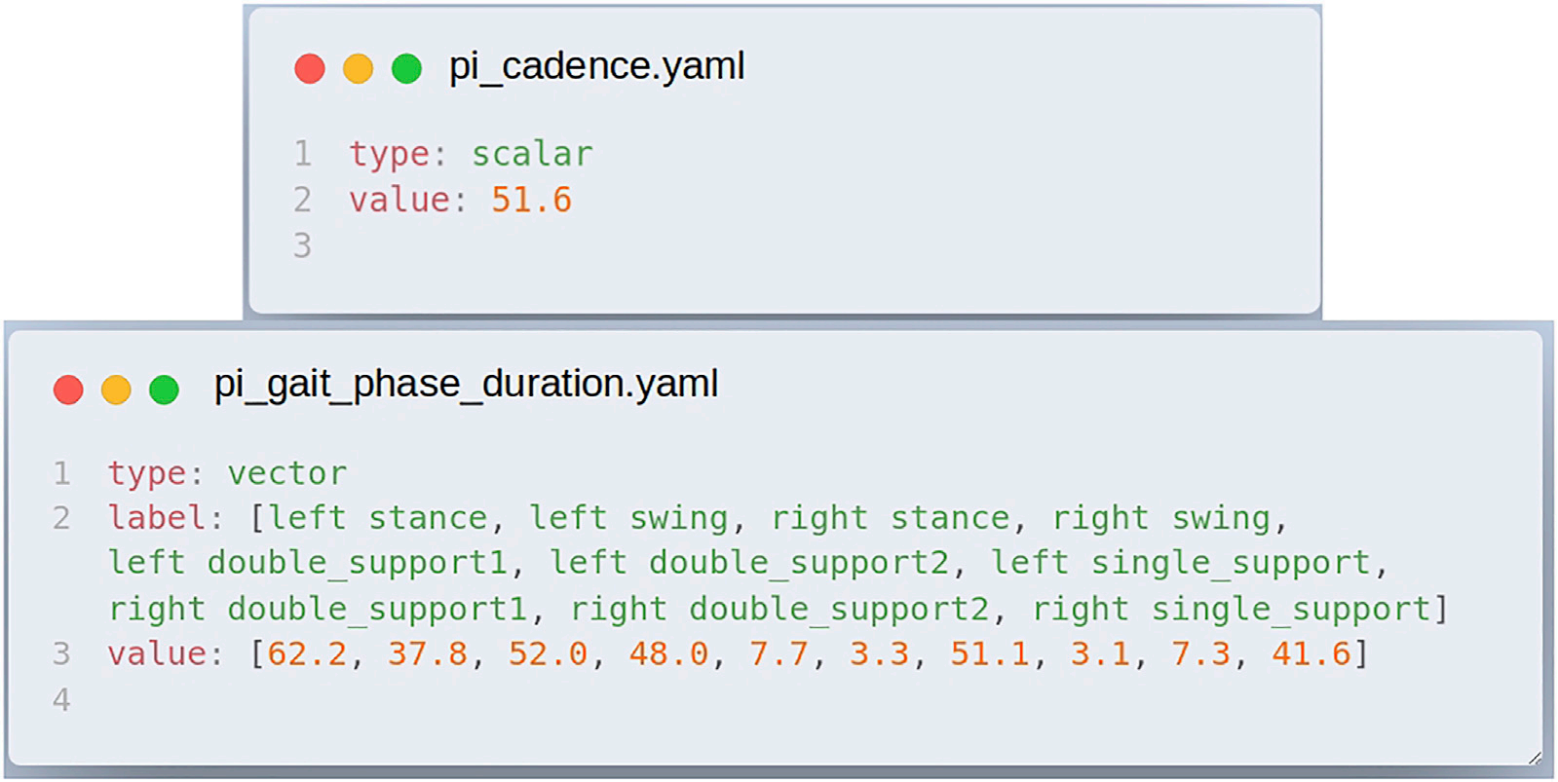
Example of performance indicators (PI) output score files, obtained with the walking on slope algorithm. Scores are stored as YAML files to ease potential post-processing.

Figure 3 provides an example of two metrics obtained with a protocol characterizing the kinematics of inclined walking (algorithm *rrd_pi_slope* in Table 1). File *pi_cadence.yaml* describes the subject cadence in steps per minute as scalar information. File *pi_gait_phase_duration.yaml* describes the duration of the main gait phases. It is stored as a vector, and labels are provided to easily relate the scores to their meanings. The complete format definition is available on the Eurobench documentation webpage (see section performance indicator of the Eurobench documentation^12^).

**TABLE 1:** List of protocol metrics following Eurobench format.

Name	Language	Protocol
Wearable-related protocols		
*rrd_pi_slope*	python	kinematics of stair walking and inclined walking
*pi_bench*	octave	sit-to-stand performance analysis
*pi_sbs_human_factor*	python	human factor study during stair walking, Maugliani et al. (2022)
*sbs_emg*	matlab	EMG signal study during stair walking, Maugliani et al. (2022)
*pi_sbs_biomechanics*	python	biomechanics analysis of stair walking, Maugliani et al. (2022)
*pi_udbenchmark*	python	walking with a prosthesis on a slope and instrumented treadmill, Bruijn et al. (2013)
*pi_pepato*	octave	EMG analysis during treadmill walking
*pi_bestable*	octave	visually cued stepping perturbations on a treadmill
*pi_ctag*	octave	industrial use case in narrow space
*pi_beat*	octave	walking/standing on a moving surface
*pi_csic_irregular*	octave	walking on irregular terrain
*pi_bullet*	octave	walking with crutches
Humanoid-related protocols		
*dysturbance*	matlab	stability analysis under external perturbation
*eb_hum_bench*	python	humanoid platform walking characterization, Aller et al. (2021)
*pi_comtest*	octave	standing on a moving surface, Lippi et al. (2022)
*pi_madrob*	python	opening/closing doors, Piazza et al. (2022)
*pi_beast*	python	walking with a trolley or walker, Piazza et al. (2020)
Illustrative algorithm templates		
*pi_octave_csic*	octave	example of octave metric
*pi_python_duration*	python	example of python metric
*pi_cpp_duration*	C++	example of C++ metric

In the Eurobench scope, we chose to focus PI computation on a single run. As stated above, this single run focus presents the advantage of simplifying the algorithm structure and interface, following the software principle of separation of concerns (Laplante, 2007). The computation of the metrics for all runs, conditions, and subjects is thus simplified and consists of launching the *atomic* PI algorithm iteratively for each of these settings. Externalizing the looping process enables narrowing the “responsibility” of the PI algorithm, which is a well-established principle in software programming for developing more robust code (Martin et al., 2003).

In a previous study (Remazeilles et al., 2022), we mentioned the need for aggregating PI to generate a score across runs, possibly across conditions and/or subjects. Such a mechanism would be appropriate for protocols aiming at comparing the subject behavior with or without the exoskeleton, or with different conditional settings, which would be related to comparing the scores obtained between conditions *C*_*x*_ and *C*_*y*_. We consider that making this aggregation after the computation of PI per run enforces the experimenter to get a lighter aggregation module, which is then easier to describe, compare and review.

### 2.4 Algorithm Containerization

The Eurobench project allowed us to collaborate with numerous technicians, engineers, and investigation centers, which helped us develop all protocols and related metric algorithms. As one can easily imagine, there is no universal consensus on the most appropriate programming language, and each contributor can be convinced that the language she/he uses is the best one, being python, octave, matlab, C++, or any other languages. Reaching an agreement on the best language is a never-ending exercise, and we considered it a better strategy to let the contributors use the environment they master to implement their metrics.

**FIGURE 4:**
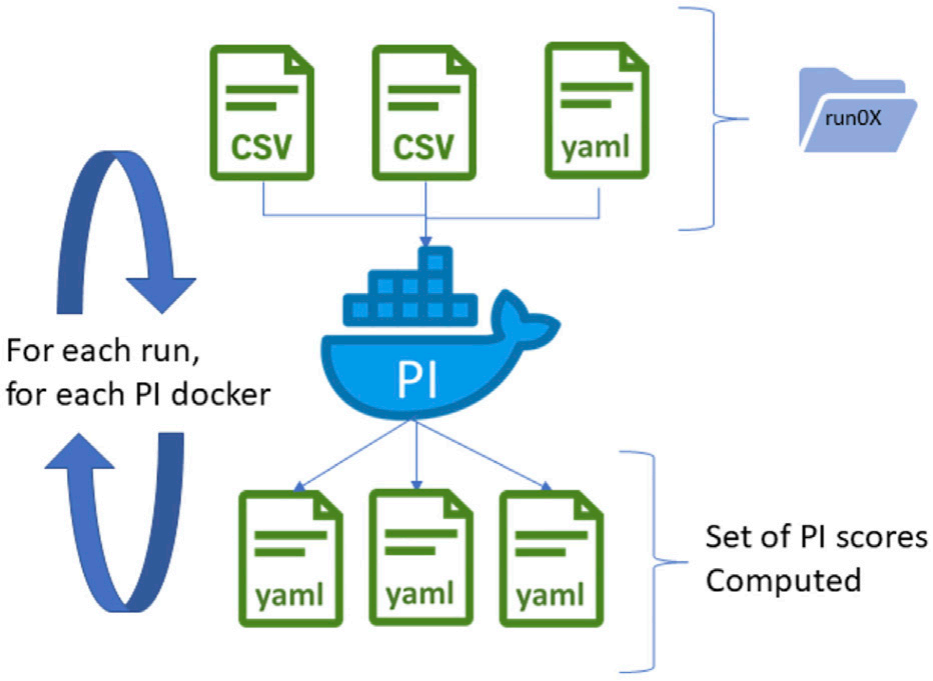
PI computation based on dockerized algorithms. Algorithms are deployed into Docker images, providing a generic interface to the algorithm independent of the programming language. The Docker container expects preprocessed datafiles of a single run as input and generates a set of PI scores in YAML files. If several runs or conditions are available, the Docker image is relaunched successively for each iteration, providing scores for each run.

To get a harmonized interface of these heterogeneous programs, we use containerization technologies based on Docker under Linux. Each scoring code is associated with a Docker file describing how to create the appropriate Docker image. The building of the code through the Docker file is much more stable as it does not rely on external dependencies that should be present on the hosting machine. In addition, this approach limits the risks of conflicts between various metric code dependencies, as all Docker images are standalone. It also provides a generic way to launch each scoring code for the central server but also for any potential users, as long as the Docker technology is installed on the deployment machine (see Figure 4).

### 2.5 Good Practices for Code Hosting

The application of this methodology, progressively designed during the project, required a learning curve and proper support from the protocol providers. We also tried to use several software tools usually associated with continuous integration and deployment (CI-CD) processes. This resulted in the following instructions to all developers:

(1) Each scoring code should be stored in a single repository under version control.
(2) A readme file should be provided indicating the purpose of the code, providing installation guidelines, examples of use, and acknowledgment of owner and funding entities.
(3) Each scoring code should be provided with reference input and associated output files to illustrate how to use it and enable regression testing.
(4) Each repository should be provided with its Docker file for generating the related Docker image.
(5) CI tools are used to verify the format of input and output files.
(6) CI tools are used to automatically generate the Docker image and test it using the reference input and output files.
(7) The Docker image is automatically deployed to a Docker hub space centralizing all these images.

Version control is nowadays a key practice to ease code maintenance, and proper documentation (readme file) is required to permit collaboration. Providing reference input and related output files with the code has various objectives. It provides examples of required input datafiles (which information is required to launch the code) and expected associated result files (which result is provided). With this reference, we can verify that the relation input file–output result is always maintained. Through the CI mechanism, we can even launch this verification automatically every time the code is changed on the git repository. In addition, the presence of the Docker image, which is generated and tested through the CI process^13^, enables any user to seamlessly install the code on her/his machine. We rely on the expertise of the code developer to target the replicability: any experiment described with similar files as required by a given protocol should be benchmarked with the metric algorithm associated with the protocol.

## 3 Results

This methodology has been applied to most of the algorithms developed in the context of the Eurobench project. All the algorithms publically available are accessible as git repositories from the Eurobench GitHub area^14^ and are listed in the following Table 1

The last three repositories are provided as examples of metrics for development performed in octave, python, and cpp, while the 17 others contain metrics associated with concrete protocols. All generated Docker images are brought to dockerhub^15^. They can thus be directly used under Linux without going through the code compilation and installation process.

## 4 Conclusion and Future Work

In this article, we have described the methodology developed within the European project Eurobench to standardize all algorithms developed for benchmarking bipedal systems. We decided to focus on the interface of the algorithm to enable developers to use their preferred language. Nevertheless, we strongly encourage reducing the algorithm scope to focus on a single metric and to focus on the processing of a single trial or run, to reduce the code complexity and ease maintenance. In terms of the input datafile, the use of simple and nonproprietary file formats is a good practice. We also suggest using algorithm input files that are preprocessed to be sensor-brand independent as it enlarges the benchmarking replicability. The use of version control and continuous integration tools is encouraged to maintain the software algorithms. In addition, providing reference datafiles with the code helps to illustrate to potential users the type of input files required to launch the code. It also permits the insertion of regression tests to ensure that the results are maintained even when the code is being changed. The dockerization of the algorithm is a convenient solution for enabling metric use without going through the installation process, which can be complex in some cases. Moreover, the Docker file used to build the container provides all the required steps to install the code in addition to the indications provided with the repository documentation.

The protocol and metrics that went through these methodologies are displayed on the project GitHub page, and examples of repository settings are provided for the most common languages, such as python, octave, C++, and matlab.

As stated in section 2.3, for simplification purposes, the PI algorithms were requested to compute metrics from a single run, letting the experimenter compare behaviors under different conditions (like comparing score outcomes under conditions *with the exoskeleton* and *without the exoskeleton*). A relevant extension of this work would be to codify and accordingly automate the comparison of the system performance under the different conditions of use, which can be involving (or not) the exoskeleton, changing its tuning, changing the environment settings, etc. Work in this direction would also enable one to define how two experiments should be compared, taking care of all the combinations that may occur. Such an aggregation process should also envision how to handle the variability of results that may be produced by the involvement of different human subjects with different capabilities and/or disabilities. Standardizing the score aggregations across runs, conditions, and subjects would also permit the consideration of generic tools for displaying the results, depending on their type and their meaning.

Limited effort has been dedicated so far to verify the code quality. The software practice of *linting* code files to verify the compliance to file format rules has been only applied to input and output datafiles (i.e., csv and yaml formats). Similar effort should be dedicated to the code itself to check and improve the code quality, which will also ease its maintenance. In addition, significant effort could be dedicated to verify the code robustness with respect to the input datafile format (i.e., can we switch two columns in a given timestamped file?), or with respect to the potential error messages (i.e., can the user understand why an error occurred, to arrange the input file format for example?).

Moreover, the current methodology does not consider yet the use of AI tools. Such an extension would permit adding reasoning capabilities across experiments, such as the correlation between device design and control choices, and the resulting metric scores. The standardization of experiment datafiles and the resulting PI scores is the first step in this direction. Meta information should be selected to describe the robotic system characteristics in terms of kinematics, control mode, etc. With this and through the accumulation of experiments following the proposed methodology, we can imagine deducing from AI possible design suggestions according to the PIs to optimize, which would be a step change in the benchmarking journey.

To conclude, it is interesting to note that the methodology presented to standardize the benchmarking process is quite generic as it focuses on defining common input files, generic PI algorithm interfaces, and scalable PI output formats. The methodology here is applied to protocols designed for exoskeleton evaluations and protocols focused on humanoid platforms. Thus, relevant future work should consider the extension to other benchmark applications to consolidate the benchmarking framework.

## Data Availability

Publicly available datasets were analyzed in this study. This data can be found here: https://github.com/eurobench/.

## Footnotes

1
https://www.cencenelec.eu/media/CEN-CENELEC/CWAs/RI/cwa17664_2021.pdf[Accessed: 20 May 2022]

2
https://www.nature.com/nprot/, [Accessed: May 20, 2022]

3
https://journals.plos.org/, [Accessed: 20 May 2022]

4
https://www.protocols.io/, [Accessed: 20 May 2022]

5
http://www.heronrobots.com/EuronGEMSig/, [Accessed: 20 May 2022]

6
https://codeocean.com/, [Accessed: 20 May 2022]

7
https://datadryad.org, [Accessed: 20 May 2022]

8
https://figshare.com, [Accessed: 20 May 2022]

9
https://zenodo.org/, [Accessed: 20 May 2022]

10
https://osf.io/, [Accessed: 20 May 2022]

11 See https://eurobench.github.io/sofware_documentation/latest/template.html

12
https://eurobench.github.io, [Accessed: 20 May 2022]

13 We developed a tool for testing code within a Docker image, verifying that given a reference input, the program effectively returns the expected output. It is used in all repositories handled in Eurobench and is accessible at https://github.com/eurobench/docker_test

14
https://github.com/eurobench, [Accessed: 20 May 2022]

15
https://hub.docker.com/u/eurobenchtest, [Accessed: 20 May 2022]

## References

[B1] Aller F. Harant M. Sontag S. Millard M. Mombaur K. (2021). “I3sa: The Increased Step Size Stability Assessment Benchmark and its Application to the Humanoid Robot Reem-C,” in 2021 IEEE/RSJ International Conference on Intelligent Robots and Systems (IROS), 5357–5363. 10.1109/IROS51168.2021.9636429

[B2] Baker M. (2016). 1,500 Scientists Lift the Lid on Reproducibility. Nature 533, 452–454. 10.1038/533452a 27225100

[B3] Bonsignorio F. (2017). A New Kind of Article for Reproducible Research in Intelligent Robotics [from the Field]. IEEE Robot. Autom. Mag. 24, 178–182. 10.1109/MRA.2017.2722918

[B4] Bonsignorio F. Hallam J. del Pobil A. (2008). Gem Guidelines: Euron Gem Sig Report Online Report.

[B5] Bruijn S. M. Meijer O. G. Beek P. J. van Dieën J. H. (2013). Assessing the Stability of Human Locomotion: a Review of Current Measures. J. R. Soc. Interface. 10, 20120999. 10.1098/rsif.2012.0999 23516062 PMC3645408

[B6] Carfì A. Foglino F. Bruno B. Mastrogiovanni F. (2019). A Multi-Sensor Dataset of Human-Human Handover. Data Brief 22, 109–117. 10.1016/j.dib.2018.11.110 30581913 PMC6297854

[B7] Fosch Villaronga E. (2018). “Legal Frame of Non-social Personal Care Robots,” in New Trends in Medical and Service Robots. Editors Husty M. Hofbaur M. (Cham: Springer International Publishing), 229–242. 10.1007/978-3-319-59972-4_17

[B8] He Y. Eguren D. Luu T. P. Contreras-Vidal J. L. (2017). Risk Management and Regulations for Lower Limb Medical Exoskeletons: A Review. Mder 10, 89–107. 10.2147/MDER.S107134 PMC5431736 28533700

[B9] Kim Y. M. Poline J. B. Dumas G. (2018). Experimenting with Reproducibility: a Case Study of Robustness in Bioinformatics. GigaScience 7. 10.1093/gigascience/giy077.Giy077 PMC6054242 29961842

[B10] Laplante P. A. (2007). What Every Engineer Should Know about Software Engineering. Boca Raton, FL: CRC Press.

[B11] Lippi V. Mergner T. Maurer C. Seel T. (2022). “Performance Indicators of Humanoid Posture Control and Balance Inspired by Human Experiments,” in Wearable Robotics: Challenges and Trends. Editors Moreno J. C. Masood J. Schneider U. Maufroy C. Pons J. L. (Cham: Springer International Publishing), 597–601. 10.1007/978-3-030-69547-7_96

[B12] Mandery C. Terlemez O. Do M. Vahrenkamp N. Asfour T. (2015). “The Kit Whole-Body Human Motion Database,” in 2015 International Conference on Advanced Robotics (Istanbul, Turkey: ICAR), 329–336. 10.1109/ICAR.2015.7251476

[B13] Martin R. C. Rabaey J. M. Chandrakasan A. P. Nikolić B. (2003). Agile Software Development: Principles, Patterns, and Practices. Upper Saddle River, NJ: Prentice-Hall.

[B14] Massardi S. Pinto-Fernandez D. F.Veneman J. J. Torricelli D. (2022). “Testing Safety of Lower Limbs Exoskeletons: Current Regulatory Gaps,” in Wearable Robotics: Challenges and Trends. Editors Moreno J. C. Masood J. Schneider U. Maufroy C. Pons J. L. (Cham: Springer International Publishing), 145–149. 10.1007/978-3-030-69547-7_24

[B15] Maugliani N. Caimmi M. Malosio M. Airoldi F. Borro D. Rosquete D. (2022). “Lower-limbs Exoskeletons Benchmark Exploiting a Stairs-Based Testbed: The Stepbystep Project,” in Wearable Robotics: Challenges and Trends. Editors Moreno J. C. Masood J. Schneider U. Maufroy C. Pons J. L. (Cham: Springer International Publishing), 603–608. 10.1007/978-3-030-69547-7_97

[B16] Maurice P. Malaisé A. Amiot C. Paris N. Richard G.-J. Rochel O. (2019). Human Movement and Ergonomics: an Industry-Oriented Dataset for Collaborative Robotics. Int. J. Robotics Res.. 10.1177/0278364919882089

[B17] Piazza E. Fontana G. Matteucci M. Nardi D. Migliavacca M. (2020). “Quantitative Evaluation of Humanoid Robots,” in In 2020 I-RIM Conference (I-RIM), 33–34. 10.5281/zenodo.4781102

[B18] Pinto-Fernandez D. Torricelli D. Sanchez-Villamanan M. d. C. Aller F. Mombaur K. Conti R. (2020). Performance Evaluation of Lower Limb Exoskeletons: A Systematic Review. IEEE Trans. Neural Syst. Rehabil. Eng. 28, 1573–1583. 10.1109/tnsre.2020.2989481 32634096

[B19] Remazeilles A. Dominguez A. Barralon P. Torricelli D. (2022). “Towards a Unified Terminology for Benchmarking Bipedal Systems,” in Wearable Robotics: Challenges and Trends. Editors Moreno J. C. Masood J. Schneider U. Maufroy C. Pons J. L. (Cham: Springer International Publishing), 609–613. 10.1007/978-3-030-69547-7_98

[B20] Riener R. (2016). The Cybathlon Promotes the Development of Assistive Technology for People with Physical Disabilities. J. Neuroeng Rehabil. 13, 49. 10.1186/s12984-016-0157-2 27246601 PMC4886429

[B21] Teytelman L. Stoliartchouk A. Kindler L. Hurwitz B. L. (2016). Protocols.io: Virtual Communities for Protocol Development and Discussion. PLoS Biol. 14, e1002538. 10.1371/journal.pbio.1002538 27547938 PMC4993360

[B22] Torricelli D. Gonzalez-Vargas J. Veneman J. F. Mombaur K. Tsagarakis N. del-Ama A. J. (2015). Benchmarking Bipedal Locomotion: A Unified Scheme for Humanoids, Wearable Robots, and Humans. IEEE Robot. Autom. Mag. 22, 103–115. 10.1109/MRA.2015.2448278

[B23] Torricelli D. Pons J. L. (2019). “Eurobench: Preparing Robots for the Real World,” in Wearable Robotics: Challenges and Trends. Editors Carrozza M. C. Micera S. Pons J. L. (Cham: Springer International Publishing), 375–378. 10.1007/978-3-030-01887-0_72

[B24] Torricelli D. Rodriguez-Guerrero C. Veneman J. F. Crea S. Briem K. Lenggenhager B. (2020). Benchmarking Wearable Robots: Challenges and Recommendations from Functional, User Experience, and Methodological Perspectives. Front. Robot. AI 7. 10.3389/frobt.2020.561774 PMC7805816 33501326

[B25] Whitaker K. (2017). Showing Your Working: A How to Guide to Reproducible Research. 10.6084/m9.figshare.4244996.v2

[B26] Wojtusch J. von Stryk O. (2015). “Humod - a Versatile and Open Database for the Investigation, Modeling and Simulation of Human Motion Dynamics on Actuation Level,” in IEEE-RAS International Conference on Humanoid Robots (Seoul, Korea (South): Humanoids), 74–79. 10.1109/HUMANOIDS.2015.7363534

